# Specific inhibition of c-Jun N-terminal kinase delays preterm labour and reduces mortality

**DOI:** 10.1530/REP-15-0258

**Published:** 2015-10

**Authors:** Grisha Pirianov, David A MacIntyre, Yun Lee, Simon N Waddington, Vasso Terzidou, Huseyin Mehmet, Phillip R Bennett

**Affiliations:** Imperial College Parturition Research Group, Department of Reproductive Biology, Institute of Reproductive and Developmental Biology, Imperial College London Hammersmith Campus, Du Cane Road, London, W12 0NN UK; Gene Transfer Technology Group, Institute for Women's Health, University College London 86–96 Chenies Mews, London, WC1E 6HX UK; Proteostasis Therapeutics 200 Technology Square Suite 402, Cambridge, Massachusetts, 02139 USA; Department of Biomedical and Forensic Sciences Anglia Ruskin University East Road, Cambridge, CB1 1PT UK

## Abstract

Preterm labour (PTL) is commonly associated with infection and/or inflammation. Lipopolysaccharide (LPS) from different bacteria can be used to independently or mutually activate Jun N-terminal kinase (JNK)/AP1- or NF-κB-driven inflammatory pathways that lead to PTL. Previous studies using *Salmonella abortus* LPS, which activates both JNK/AP-1 and NF-κB, showed that selective inhibition of NF-κB delays labour and improves pup outcome. Where labour is induced using *Escherichia coli* LPS (O111), which upregulates JNK/AP-1 but not NF-κB, inhibition of JNK/AP-1 activation also delays labour. In this study, to determine the potential role of JNK as a therapeutic target in PTL, we investigated the specific contribution of JNK signalling to *S. Abortus* LPS-induced PTL in mice. Intrauterine administration of *S. Abortus* LPS to pregnant mice resulted in the activation of JNK in the maternal uterus and fetal brain, upregulation of pro-inflammatory proteins COX-2, CXCL1, and CCL2, phosphorylation of cPLA2 in myometrium, and induction of PTL. Specific inhibition of JNK by co-administration of specific D-JNK inhibitory peptide (D-JNKI) delayed LPS-induced preterm delivery and reduced fetal mortality. This is associated with inhibition of myometrial cPLA2 phosphorylation and proinflammatory proteins synthesis. In addition, we report that D-JNKI inhibits the activation of JNK/JNK3 and caspase-3, which are important mediators of neural cell death in the neonatal brain. Our data demonstrate that specific inhibition of TLR4-activated JNK signalling pathways has potential as a therapeutic approach in the management of infection/inflammation-associated PTL and prevention of the associated detrimental effects to the neonatal brain.

## Introduction

Globally, more than 15 million babies are born preterm each year (Blencowe *et al*. 2013), of which around 1 million die from associated complications. Many survivors experience lifetime learning disabilities and visual and hearing problems. The risk of cerebral palsy (CP) is increased 70-fold in infants born <28 weeks gestation and some 90% of infants born before 30 weeks gestation show brain abnormalities on MRI when imaged at term-corrected age (Romero *et al*. 2006). Intrauterine infection and/or inflammation plays an important aetiological role in early preterm delivery and is a risk factor for subsequent CP in both term and preterm infants (Wu & Colford 2000).

Inflammation represents a common biochemical pathway critically involved in both term and preterm labour (PTL; Romero *et al*. 2006). To study the underlying biochemistry of inflammation-associated PTL, several investigators have developed mouse models based on intrauterine or systemic injection of bacteria or bacterial lipopolysaccharide (Hirsch *et al*. 1995, Kaga *et al*. 1996, Elovitz *et al*. 2003, Elovitz & Mrinalini 2004). Bacterial products induce PTL through interaction with toll-like receptor-4 (TLR4) and subsequent activation of pro-inflammatory and pro-contractile pathways within the uterus. Studies using TLR4 mutant mice show that TLR4 plays an essential role in LPS-induced PTL (Elovitz *et al*. 2003, Wang & Hirsch 2003). Activation of the TLR4 signalling pathway by LPS leads to the upregulation of prostaglandin (PG) synthesis and production of proinflammatory cytokines (Gravett *et al*. 1994, Pollard & Mitchell 1996, Elovitz *et al*. 2006). PG E2 and PGF2 and cytokines induce cervical ripening (Kelly 2002) and stimulate uterine contractions (Baggia *et al*. 1996). There is now good evidence that in addition to inducing preterm delivery, proinflammatory cytokines also mediate antenatal brain injury (Bell *et al*. 2004, Elovitz *et al*. 2006). In animal models of prenatal brain injury, injection of LPS or intact bacteria causes lesions in white matter of the neonatal brain (Debillon *et al*. 2000), including both periventricular leukomalacia (PVL), a pathology associated with the development of CP (Drougia *et al*. 2007), and diffuse white matter injury, a brain abnormality more commonly associated with adverse neurodevelopmental outcome (Dyet *et al*. 2006). Due to the association between inflammation, the onset of labour and the risk of perinatal brain injury, attention is being focused on anti-inflammatory agents as novel therapeutic options to prevent PTL (Rinaldi *et al*. 2011, MacIntyre *et al*. 2012, Sykes *et al*. 2014).

Animal studies have demonstrated that several anti-inflammatory agents delay preterm delivery and improve pup survival, including cytokine IL-10 (Terrone *et al*. 2001, Rodts-Palenik *et al*. 2004), short-chain fatty acids (Voltolini *et al*. 2012) and lipoxins (Macdonald *et al*. 2011). The transcription factor nuclear factor kappa B (NF-κB) plays a pivotal role in the upregulation of pro-labour and pro-inflammatory genes associated with parturition (Choi *et al*. 2007) and is proposed to inhibit progesterone receptor function and thus block uterine quiescence (Condon *et al*. 2006). We, and others, have shown that NF-κB inhibition leads to decreased synthesis of cytokines that trigger both preterm delivery and neonatal brain injury, making it an attractive therapeutic target in the management of PTL (Adams Waldorf *et al*. 2008, Pirianov *et al*. 2009, Li *et al*. 2010, Nath *et al*. 2010).

We have recently shown that normal labour onset in the mouse involves the sequential activation of the transcription factors NF-κB and AP-1 within the uterus (MacIntyre *et al*. 2014). Additionally, we have identified differential activation of NF-κB and Jun N-terminal kinase (JNK) in two mouse models of LPS-induced PTL (Pirianov *et al*. 2009, MacIntyre *et al*. 2014). PTL induced using highly TRL4-specific *Salmonella abortus* LPS activates both NF-κB and JNK and leads to the upregulation of cPLA2 and COX-2, both central to prostaglandin synthesis, as well as the stimulation of labour-associated cytokines, CCL-2 and CXCL-1, in the myometrium (Pirianov *et al*. 2009). Inhibition of NF-κB activity, JNK activity and cytokine synthesis by the anti-inflammatory cyclopentenone prostaglandin 15-deoxy-δ12, 14-prostaglandin J2 (15d-PGJ2), delays preterm birth and improves pup survival. In contrast, induction of PTL by *Escherichia coli*-derived LPS involves AP-1 activation via JNK, but does not involve NF-κB activation (MacIntyre *et al*. 2014). In this model, inhibition of JNK using SP600125 delays PTL. Where labour is induced using the PR/GR antagonist RU486, no sequential activation of NF-κB and AP-1 is detected in the uterus, but labour itself is associated with increased JNK activity. Thus NF-κB activation appears to be a feature of normal labour and of labour induced by some LPS serotypes, but it is not a universal feature of all models of inflammation- or non-inflammation-induced PTL.

JNK-mediated activation of AP-1 appears to be common to both inflammation- or non-inflammation-induced PTL. Further, brain activation of JNK has been shown to play a key role in perinatal brain injury. Three mammalian JNK genes (JNK1, JNK2 and JNK3) have been identified of which JNK3 is the form most predominantly active in the brain. In a mouse model where JNK3 expression was ablated, pup brain levels of c-Jun were reduced compared to wild-type (WT) animals and led to partial protection against hypoxic–ischaemic injury through a reduction of caspase-3 activation (Pirianov *et al*. 2007).

Specific inhibition of JNK, in the context of inflammation-induced PTL, has the potential to both delay delivery and improve pup outcome. However, where inflammation activates both NF-κB and JNK, specific inhibition of JNK may be insufficient to either delay preterm birth or improve pup outcome. In this study *S. abortus* LPS was used to activate both uterine NF-κB and JNK activation and cause preterm birth. Animals were then treated with a highly specific JNK inhibitor, D-JNK inhibitory peptide (D-JNKI), to determine if *in vivo* inhibition of myometrial JNK-activation and downstream inflammatory mediators such as COX-2, cPLA and cytokines delays LPS-induced preterm delivery, improves neonatal mortality and reduces JNK3 activation and thus damaging the neonatal brain.

## Materials and methods

### Reagents and antibodies

Antibodies against serine 505-phosphorylated cPLA_2_, HRP-conjugated secondary antibodies and JNK *in vitro* kinase kit and cleaved caspase-3 antibody were purchased from Cell Signaling Technology (Danvers, MA, USA). COX-2, CCL2, CXCL1 and JNK2 antibodies were purchased from Santa Cruz Biotechnology. JNK1 and JNK1/2 antibodies were obtained from Pharmingen (San Jose, CA, USA). The antibody against β-actin was from Abcam (Cambridge, UK). LPS (TLR-4 grade S form from *Salmonella abortus*) was purchased from Enzo Biosciences (Nottingham, UK). D-JNKI was kindly provided by Dr H Mehmet (Merck, Kenilworth, NJ, USA).

### Mouse model of intrauterine inflammation

All animal experiments were approved by the Imperial College London Ethical Review Board and conformed to the British Home Office regulations. CD-1 outbred mice were used in this study. This strain is commonly used in LPS-mediated models of PTL and inflammation more generally and does not exhibit any known LPS resistance. CD-1 outbred, timed-pregnant mice were obtained from Harlan Laboratories (Bicester, UK) on gestation day 13 after mating and were acclimatised for 3 days before being used in experiments. Surgery was performed on day 16 of gestation. Dams were anaesthetised by isofluorane, the uterine horns exteriorised following laparotomy and kept moist with sterile PBS. The uterine horn containing the greatest number of viable fetuses was selected for injection. A 25 μl volume LPS (1.0 μg) alone, 5 μg D-JNKI, or 5 μg LPS plus 5 μg D-JNKI was injected into the lumen of the uterus between the first and second anterior fetuses using a 33-gauge Hamilton syringe, taking care not to enter the amniotic cavity. The wound was closed in two layers. Mice received Vetalar analgesia (Parke Davis & Co. Ltd., Eastleigh, UK) and were recovered in a warm environment. For observations, each mouse was housed separately. Control animals received no anaesthesia or surgery, and sham animals received anaesthesia and an intrauterine injection of 25 μl of saline. The time from surgery to delivery was recorded, with delivery of the newborn pups and their survival rate monitored every 6 h. Animals studies were in accordance with UK Home Office licence conditions.

### Tissue harvesting and processing

Maternal uterine tissue (myometrium) and foetal brain were collected close to the injection site in the uterine horn 1 and 6 h post-injection with LPS ±D-JNKI (1, 2 or 5 μg) or vehicle control. Samples were flash frozen in liquid nitrogen and stored at −80 °C. Tissues were lysed by sonication in a non-denaturing phosphate lysis buffer consisting of 20 mM sodium phosphate, 137 mM NaCl, 25 mM sodium β-glycerophosphate, 2 mM sodium pyrophosphate, 2 mM EDTA, 10% glycerol, 1% Triton X-100 and protease inhibitor cocktail (Sigma–Aldrich). Cell lysates were incubated on ice for 20 min and centrifuged for 20 min at 12 000 ***g*** at 4 °C. Protein concentration was determined by the bicinchoninic acid method (Pierce, Rockford, IL, USA).

### RT-PCR

Total RNA was isolated using RNA STAT-60 (Tel-Test, Inc., Friendswood, TX, USA) according to the manufacturer's instructions. A total of 1 μg RNA was used as a template for reverse transcription. Expression of OTR, COX-1, COX-2, connexin 26 and 43, CCL2, CXCL-1 and GAPDH were assessed by real-time RT-PCR using an ABI PRISM 7700 Sequence Detection System according to the manufacturer's protocol (Applied Biosystems/Life Technologies). Taqman primers and probes were designed using the primer express programme (Applied Biosystems/Life Technologies). The data were analysed using Sequence Detector version 1.7 Software (Applied Biosystems/Life Technologies) and were normalised to GAPDH.

### SDS–PAGE and immunoblotting

Tissue lysates (50 μg) were separated on a 10% SDS–PAGE gel and transferred to PVDF membranes (Millipore, Billerica, MA, USA) and blocked using 5% (w/v) skimmed milk in Tris-buffered saline (TBS) supplemented with 0.1% (v/v) Tween-20 (TBST) for 1 h at room temperature. Blots were incubated overnight at 4 °C with primary antibody (1:1000 dilutions in TBS, 1% milk). After washing in TBST, blots were incubated with HRP-conjugated goat anti-rabbit antibody or rabbit anti-mouse antibody at room temperature for 1 h in 5% milk prepared in TBST. Following the final wash, immune-reactive bands were visualized on film using a chemiluminescent substrate (ECL Plus, GE Healthcare and Buckinghamshire, UK). Densitometric analysis was performed using 1D Kodak Digital Science Software (Kodak). The levels of cellular β-actin were used as a loading control.

### Immunoprecipitation of cleaved caspase-3

Tissue lysates (200 μg protein) were incubated overnight at 4 °C with G agarose beads (GE Healthcare) pre-bound with cleaved p17 caspase-3 antibody. After washing, the beads were resuspended in a sample loading buffer and heated for 5 min at 95 °C and spun down. Samples (50 μl) were separated by electrophoresis on a 14% SDS–PAGE gel, transferred to a PVDF membrane and finally incubated with cleaved caspase-3 antibody (1:1000) overnight at 4 °C following the western blotting procedure previously described.

### *In vitro* kinase assay for JNK

JNK activity was measured using a specific kit (Cell Signaling Technology) following the manufacture's instructions and using GST-Jun (1–79) fusion peptide as the specific substrate for JNK. In brief, tissue lysates (100 μg protein) were incubated overnight at 4 °C with GST-Jun fusion protein beads. After washing, the beads were resuspended in kinase buffer containing ATP and the kinase reaction was allowed to proceed for 30 min at 30 °C. The reaction was stopped by the addition of a sample loading buffer. Proteins were separated by electrophoresis on a 10% SDS–PAGE gel, transferred to a PVDF membrane and finally incubated with phospho-c-Jun (Ser63) and c-Jun antibodies. Finally, blots were subjected to enhanced chemiluminescence and kinase activity determined by densitometric analysis.

### *In vitro* kinase assay for JNK3

Currently, specific non-cross-reactive antibodies for the major JNK isoforms 1, 2 and 3 are not available. Therefore we used an approach for capturing JNK3 following immunodepletion of JNK1 and JNK2 isoforms. To specifically determine the presence of the active JNK3 isoform, cell lysates were first immunoprecipitated with a mixture of JNK1 (cross-reacts with JNK1 and 2 but not with JNK3; data not shown) and JNK2 (cross-reacts with JNK2 and 1 but not with JNK3; data not shown) antibodies already pre-bound to Protein-G beads to remove both JNK1 and JNK2 from the lysates. This process was repeated twice to ensure that JNK1 and JNK2 were completely removed from the supernatant. Depletion of JNK1 and JNK2 was confirmed by western blot analysis of cell lysates before and after immunodepletion using a JNK1/2 antibody that recognizes both the JNK1 and JNK2 isoforms. The residual active JNK3 isoform in the supernatant was subjected to *in vitro* kinase assay to measure JNK3 activity, as described above.

### Statistical analysis

Biochemical and molecular data are reported as mean±s.d. and analysed with one-way ANOVA followed by the Bonferroni post-test for multiple comparisons using GraphPad Prism version 4.0. The differences between multiple groups in PTL model were analysed by Kruskal-Wallis equality-of-population rank test.

## Results

### D-JNKI supresses LPS-induced JNK activation in mouse myometrium and fetal brain

**Figure 1:**
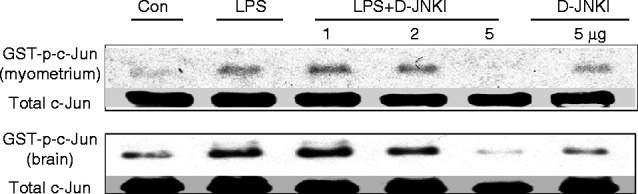
D-JNKI inhibits LPS-induced JNK activation in myometrium and neonatal brain. Timed pregnant CD-1 mice were injected on day 16 with *Salmonella abortus* LPS (1.0 μg) alone, LPS together with (1, 2 or 5 μg) of D-JNKI or with D-JNKI (5 μg) alone. Tissue samples from myometrium and fetal brain were isolated at 1 h post-treated and examined for JNK activity using an *in vitro* kinase assay. Maximal inhibition of LPS-induced JNK activation was achieved with 5 μg D-JNKI.

**Figure 2:**
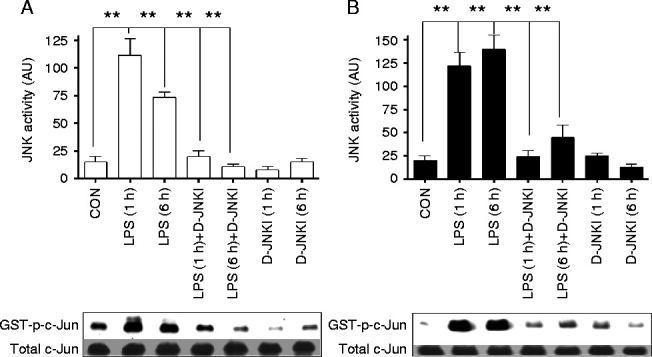
D-JNKI inhibits LPS-induced JNK activation in myometrium and neonatal brain at 1 and 6 h post-injection. Timed pregnant CD-1 mice were injected on gestation day 16 with *Salmonella abortus* LPS (1.0 μg) alone, LPS together with (5 μg) of D-JNKI or with D-JNKI (5 μg) alone. (A) Tissue samples from myometrium and (B) fetal brain were isolated at 1 and 6 h post-injection and analysed for JNK activity using an *in vitro* kinase assay. (*n*=3 **P<0.001 compared to LPS alone at each time point ANOVA).

We have previously shown that the administration of *S. abortus* LPS activates TLR4/JNK signalling in an experimental model of PTL (Pirianov *et al*. 2009). Here, the effects of D-JNKI (a specific inhibitor of JNK) on PTL were investigated. In the first series of experiments we determined the ability of co-administration of D-JNKI (0–5 μg) to block *S. abortus* LPS-induced TLR4/JNK activation in the myometrium and in the fetal brain in this mouse model of PTL. Animals were sacrificed 1 h post-injection of LPS. Because D-JNKI prevents JNK/substrate interactions but not JNK phosphorylation, we utilised an *in vitro* kinase assay to monitor the inhibitory effects of D-JNKI in tissue lysates. Kinase assay data showed that LPS injection induced JNK activity at 1 h post-injection in both the maternal myometrium and the neonatal brain. Inhibition of LPS-induced JNK activity was achieved by D-JNKI at an injection dose of 5 μg (Fig. 1A). Although this single western blot gives the impression that JNK is activated at a higher level in the D-JNKI 5 μg treated samples compared to controls and LPS+D-JNKI treated samples, quantification of three separate experiments (Fig. 2A and B) shows that D-JNKI at 5 μg has no effect on JNK activity. Based on this result, JNK activity assays were performed using 5 μg of D-JNKI as shown in Fig. 2. This dose of D-JNKI was sufficient to inhibit JNK activity up to 6 h post-injection in both the myometrium and neonatal brain following LPS injection (Fig. 2A and B).

### D-JNKI delays LPS-induced preterm delivery

**Figure 3:**
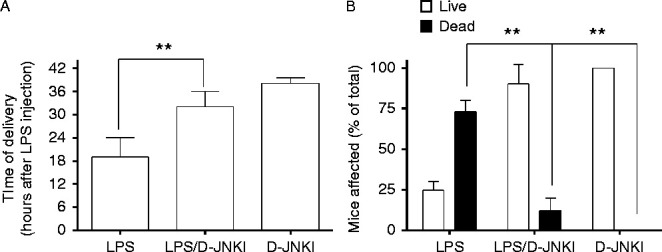
D-JNKI delays LPS-induced preterm delivery and increases pup survival. Timed pregnant CD-1 mice were injected on gestation day 16 with 1.0 μg *Salmonella abortus* LPS (*n*=7) or LPS together with 5 μg of D-JNKI (*n*=*8*) or with 5 μg of D-JNKI alone (*n*=*8*). Delivery of the newborn pups and their survival rate was monitored every 6 h. D-JNKI led to a significant increase in time to delivery as well as increased pup survival. Data represent the mean (±s.e.m.) of *n* animals at each group. **P<0.01, Kruskal-Wallis equality-of-population rank test.

In the next experiments we determined whether specific inhibition of JNK by D-JNKI would lead to delayed *S. abortus* LPS-induced PTL in this animal model. Animals were injected on gestation day 16 with *S. abortus* alone or together with 5 μg of D-JNKI or with 5 μg of D-JNKI alone. The pregnancy was monitored at 6 h intervals until delivery. *S. abortus* LPS alone caused preterm delivery after ∼20 h (±s.e.m.) (Fig. 3A) with 70% pup mortality (Fig. 3B). Co-injection of 5 μg of D-JNKI delayed preterm delivery by an average of 10 h (to a mean of 32 h; Fig. 3A) and reduced pup mortality to 10% (Fig. 3B). Injection of 5 μg of D-JNKI alone was associated with later preterm delivery at 40 h, with no pup mortality. These data show that the specific inhibition of JNK delays *S. abortus* LPS-induced PTL and increases pup survival.

### D-JNKI inhibits both LPS-induced cPLA_2_ phosphorylation and upregulation of proinflammatory proteins COX-2, CCL2 and CXCL1 in mouse myometrium

**Figure 4:**
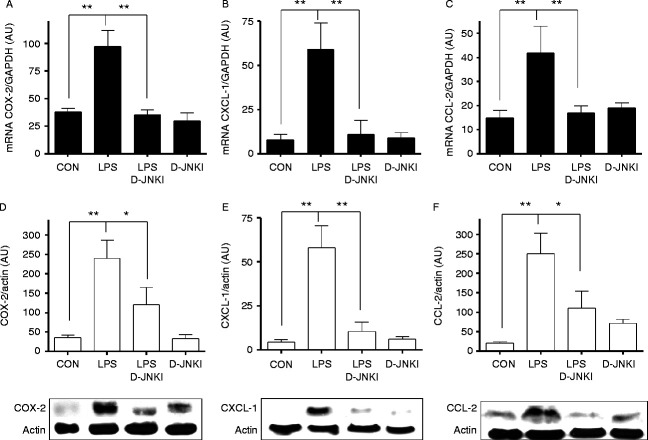
D-JNKI inhibits LPS-induced cPLA2 phosphorylation and production of COX-2, CCL-2 and CXCL-1 in myometrium. Timed pregnant CD-1 mice were injected on day 16 with 1.0 μg of *Salmonella abortus* LPS alone, LPS together with 5 μg of D-JNKI or with 5 μg of D-JNKI alone. Tissue samples from the myometrium were collected at 6 h and analysed for mRNA and protein levels of COX-2 (A and D), CCL-2 (B and E) and CXCL-1 (C and F) expression and normalised to loading controls (GAPDH and actin) as arbitrary units (AU). Data represent the mean (±s.e.m.) of three animals at each data point. The difference between LPS alone or together with D-JNKI was statistically significant by ANOVA *P<0.05 and **P<0.01.

**Figure 5:**
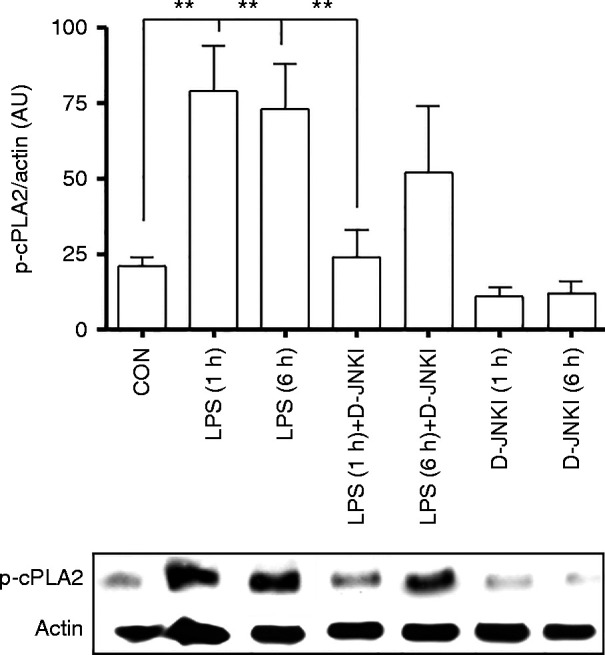
D-JNKI inhibits LPS-induced cPLA2 phosphorylation. Timed pregnant CD-1 mice were treated on gestation day 16 with *Salmonella abortus* LPS (1.0 μg) alone, LPS together with 5 μg of D-JNKI, or with 5 μg of D-JNKI alone. Tissue samples from the myometrium were prepared at 6 h and analysed for cPLA2 phosphorylation and normalised to loading control actin as arbitrary units (AU). Data represent the mean (±s.e.m.) of three animals at each data point. The difference between LPS alone or together with D-JNKI was statistically significant by ANOVA (**P<0.01).

We have previously reported that PTL induced in a mouse model using *S. abortus* LPS has no effect on OTR, connexins 23 and 46 or COX-1 but significantly upregulates mRNA levels of COX-2, CCL2 and CXCL1 and increases phosphorylation of cPLA2 in the myometrium 6 h post-injection. We therefore studied the effect of D-JNKI on *S. abortus* LPS-induced expression of COX-2, CCL2 and CXCL1 and cPLA2 phosphorylation. Upregulation of COX-2, CCL2 and CXCL1 were significantly suppressed by co-administration of D-JNKI (Fig. 4A, B and C). Using western blotting analysis, we validated the mRNA data at the protein level COX-2, CCL2 and CXCL1 in mouse myometrium (Fig. 4D and E). *S. abortus* LPS injection induced cPLA_2_ phosphorylation in maternal myometrium, which was inhibited by D-JNKI at 1 h but not after 6 h following injection (Fig. 5).

### D-JNKI supresses LPS-induced JNK3 and caspase-3 activation in the fetal brain

**Figure 6:**
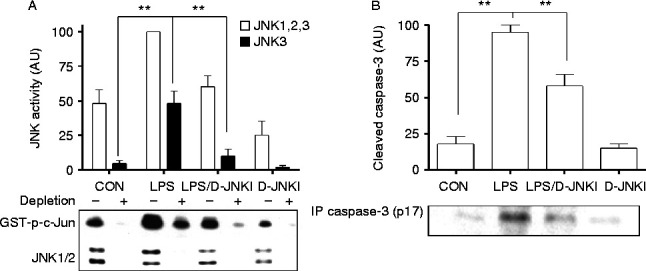
D-JNKI inhibits LPS-induced JNK/JNK3 and caspase-3 activation in the fetal brain. Pup brain extracts collected at 6 h following injection with 1.0 μg of *Salmonella abortus* LPS alone, LPS together with 5 μg of D-JNKI, or with 5 μg of D-JNKI alone were analysed for (A) JNK/JNK3 and (B) cleaved caspase-3 activation. Data represent the mean (±s.e.m.) of three animals at each data point. The difference between LPS alone or together with D-JNKI was statistically significant by ANOVA (**P<0.05).

We have previously shown that the JNK3 isoform plays a critical role in hypoxia-ischaemia-induced neural cell death and neonatal brain injury (Pirianov *et al*. 2007). We therefore examined the effect of D-JNKI on *S. abortus* LPS-driven JNK3 and caspase-3 activation in the fetal brain. Using an *in vitro* immunodepletion kinase assay (Pirianov *et al*. 2006) to measure JNK/JNK3 activity, we found that co-injection of D-JNKI downregulates JNK activation and completely inhibits JNK3 activity in the fetal brain (Fig. 6A). This effect of D-JNKI is associated with a decrease in *S. abortus* LPS-induced caspase-3 activity, measured as a production of cleaved p17 caspase-3 active fragment (Fig. 6B).

## Discussion

There exists a spectrum of severity of inflammation associated with human PTL. Overt clinical signs of chorioamnionitis are rarely seen during pregnancy in women who go on to experience an infection-associated PTL. In most cases, evidence of chorioamnionitis is only obtained following histological examination of the placenta and membranes following delivery. We originally developed the mouse model of inflammation-associated preterm delivery described in this study to model the clinical presentation in humans. The model is based on those developed by Hirsch *et al*. (1995) and Elovitz *et al*. (2003, 2006), in which sonicated bacteria or *E. coli*-derived LPS are used to induce preterm birth. However, these models report high variations in the LPS dose required, time to delivery and level of pup mortality. Our *S. abortus* LPS model has the advantage of reliable preterm delivery rates and controllable rates of pup mortality thus permitting *in vivo* study more representative of human presentation.

LPS derived from different bacterial species are known to differentially induce TLR4 signalling pathways (Park & Lee 2013). However, it is increasingly recognised that LPS molecules from the same bacterial species belonging to different serotypes also differentially interact and stimulate the TLR4 receptor (Chessa *et al*. 2014). We have previously reported that LPS isolated from either *S. abortus *or* E. coli* will activate PTL in the mouse, yet time to delivery and pup survival rates vary significantly (Pirianov *et al*. 2009, MacIntyre *et al*. 2014). Moreover, we have shown that *E. coli*-derived LPS serotypes differentially activate inflammatory pathways in the mouse myometrial and pup brain leading to major differences in maternal and fetal outcomes (unpublished data). While all four of the *E. coli* LPS serotypes tested led to preterm birth, time to preterm delivery onset was strongly correlated with the level of JNK/AP1 activation but not correlated with NF-κB activation. Recent studies using *E. coli* serotype O111 (B4) alone have shown that preterm delivery in the mouse involves myometrial activation of AP-1 via JNK without upregulation of NF-κB activity and that inhibition of JNK can delay PTL (MacIntyre *et al*. 2014). In contrast, *S. abortus *LPS leads to preterm delivery that is associated with myometrial activation of both NF-κB and JNK/AP-1 and that inhibition of NF-κB by the cyclo-pentenone 15d-PGJ2 delays preterm delivery, improves pup survival and inhibits myometrial and brain inflammation in the mouse PTL model primarily (Pirianov *et al*. 2009). The relative contribution of NF-κB and JNK/AP-1 to both PTL and fetal outcomes is yet to be fully elucidated. Thus, in this study we set out to determine whether specific inhibition of JNK would also be effective in a model in which both NF-κB and JNK/AP-1 are activated. We selected the D-JNKI rather than SP600125 because the latter is a comparably weak and non-specific inhibitor of JNK (Jin *et al*. 2009). The D-JNKI used in this study is a small peptide pharmacological of JNK activity based on specific interaction with substrate binding domain of JNK (Bonny *et al*. 2001, Barr *et al*. 2002). D-JNKI is a highly cell permeable, decoy substrate, which potently and selectively inhibits all JNK isoforms and has the potential to be used as a small molecule drug.

In the initial dose response studies, animals were sacrificed at 1 h after injection of LPS. This time point was chosen for the harvesting of uterine and brain samples because JNK is thought to be involved in the early signal transduction pathways leading to labour onset and neonatal brain injury, and previous work has shown that cytokine levels in the mouse uterus and brain are elevated within 1–8 h of LPS administration (Hirsch *et al*. 1995, Elovitz *et al*. 2006). Accordingly, effective and clinically relevant strategies for JNK inhibition should target the early phase of inflammatory pathway induction. Once the suitable dose of D-JNKI had been determined, the effect of that dose at early (1 h) and later (6 h) times points was confirmed (Fig. 1B). As D-JNKI prevents JNK/substrate interactions but not JNK phosphorylation, we then utilised an *in vitro* kinase assay, rather than a marker of JNK phosphorylation (indirect JNK activation), to monitor the inhibitory effects of D-JNKI in myometrium and brain tissue lysates (Fig. 2A and B). Significant inhibition of JNK activity by D-JNKI was also achieved at both 1 and 6 h time points indicating that D-JNKI possesses a rapid and direct blockage of JNK/AP1 activity that is sustained for at least 6 h in an *in vivo* model of infection-induced preterm birth.

Consistent with our previous findings, *S. abortus* LPS was shown to induce PTL onset 18–24 h post-treatment with 70% pup mortality (Pirianov *et al*. 2009) (Fig. 3). Co-injection of 5 μg of D-JNKI delayed preterm delivery until 32 h post-injection and reduced pup mortality to 10%. Therefore, inhibition of JNK/AP1 by D-JNKI delays preterm delivery and improves pup mortality to a similar extent as inhibition of NF-κB by the cyclo-pentenone 15d-PGJ2, when tested in the same mouse model of preterm birth (Pirianov *et al*. 2009). There were no major differences between the ability of D-JNKI or 15d-PGJ2 to delay labour or improve pup outcome suggesting that a strategy of directly targeting JNK rather than NF-κB will be effective whether the inflammatory stimulus does or does not activate NF-κB.

In addition to inhibition of inflammatory transcription activation, we also showed that D-JNKI significantly reduced mRNA and protein levels of various downstream pro-labour and pro-contractile mediators including CCL2, CXCL1 and COX-2 when assessed at 6 h post-LPS injection (Fig. 4). Moreover, inhibition of enzyme cPLA2, which catalyses the release of arachidonic acid from membrane phospholipids, was achieved by D-JNKI at 1 h compared with LPS-alone treated animals. While the ERK1/2 (p44/p42) or p38 isoforms of MAPK have historically been considered as the primary kinases responsible for cPLA2 serine 505 phosphorylation (Leslie 1997, Ghosh *et al*. 2006), JNK has recently been identified as the key mediator of cPLA2 serine 505 phosphorylation and subsequent translocation to the membrane in human macrophages (Casas *et al*. 2009), suggesting that these events are likely to be cell type and activation stimuli dependent. In support of the latter, we show that D-JNKI inhibits early phosphorylation of cPLA2 at 1 h post-treatment but not at the later 6 h time point. This raises the possibility that cPLA2 phosphorylation in the myometrium is modulated in a phasic manner by multiple kinases.

The typical length of gestation in these animals is 18.5 days. We found that D-JNKI alone had a modest impact on reducing gestation length. It did not, however, have any effect on CCL-2, CXCL-1 or COX-2 expression. This suggests that D-JNKI alone did not lead to early changes in the inflammatory response. Why D-JNKI alone caused slight preterm delivery is unclear, but, importantly, pup mortality rates were similar in the D-JNKI and injected control group. D-JNKI does inhibit all three isoforms of JNK and we cannot exclude the possibility that one of these isoforms is involved in the regulation of parturition at term. JNK has been shown to play a key role in neonatal hypoxic–ischaemic brain injury (Dreskin *et al*. 2001). While JNK1 and JNK2 are widely expressed in a range of tissues, expression of JNK3 is largely confined to the brain and testis (Kyriakis *et al*. 1994). Gene ablation studies have shown that JNK1/JNK2 are essential for normal development while deletion of JNK3 results in apparently normal animals (Kuan *et al*. 2003). However, JNK3 knockout mice are resistant to excitotoxic injury (Yang *et al*. 1997) and partially resistant to experimental hypoxic–ischaemic brain injury (Kuan *et al*. 2003). This resistance to hypoxic–ischaemic brain injury applies to JNK3 knockout neonatal mice in which absence of JNK3 significantly truncates hypoxic–ischaemic induction of caspase-3 and the associated apoptosis (Pirianov *et al*. 2007). Fetal LPS exposure via the intrauterine route alone has been shown to cause a reduction in oligodendrocyte or myelin markers without macroscopic lesions being evident. However, fetal LPS exposure via the intrauterine route sensitises the brain to subsequent hypoxic–ischaemic brain injury. We therefore hypothesised that JNK3 activation may be unregulated in the brain following LPS exposure via the intrauterine route. We found that *S. abortus* LPS leads to the upregulation of overall JNK activity in the brain. Using an immunodepletion assay in which JNK1 and JNK2 are removed, we showed that LPS injection causes a large increase in JNK3 activity, increasing its contribution to total JNK activity to 50%. This is associated with increased caspase-3 activity. Observed increases in both JNK3 and caspase-3 activity in response to LPS were significantly inhibited by D-JNKI (Fig. 6), suggesting that this small molecule has a neuroprotective function.

In mouse models of infection/inflammation-induced PTL, activation of JNK is a common final pathway leading to parturition independent of the serotype of LPS used to induce labour. Specific inhibition of JNK prolongs pregnancy, improves neonatal pup death rates and reduces the expression of inflammatory mediators within the myometrium. LPS exposure of the fetus via the intrauterine injection route activates JNK3 and caspase-3, known mediators of neuronal cell death. This activation is also inhibited by D-JNKI. The data that we present here shows that inhibition of JNK improves immediate pup survival rates and reduces JNK-3 neurotoxic activity in the brain. Whether this will in fact lead to measurable improvements in outcomes of PTL and neonatal cerebral injury requires further investigation. However, a small molecule inhibition of JNK represents an attractive anti-inflammatory strategy in the management of PTL and its associated neonatal cerebral injury.

## Declaration of interests

The authors declare that there is no conflict of interest that could be perceived as prejudicing the impartiality of the research reported.
